# PP2A inhibitors suppress migration and growth of PANC-1 pancreatic cancer cells through inhibition on the Wnt/β-catenin pathway by phosphorylation and degradation of β-catenin

**DOI:** 10.3892/or.2014.3266

**Published:** 2014-06-13

**Authors:** MENG-YAO WU, XIN XIE, ZE-KUAN XU, LI XIE, ZHENG CHEN, LIU-MEI SHOU, FEI-RAN GONG, YU-FENG XIE, WEI LI, MIN TAO

**Affiliations:** 1 Department of Oncology, The First Affiliated Hospital of Soochow University, Suzhou, Jiangsu 215006, P.R. China; 2 Department of General Surgery, The First Affiliated Hospital of Nanjing Medical University, Nanjing, Jiangsu 210029, P.R. China; 3 Department of Hematology, The First Affiliated Hospital of Soochow University, Suzhou, Jiangsu 215006, P.R. China; 4 Jiangsu Institute of Hematology, The First Affiliated Hospital of Soochow University, Suzhou, Jiangsu 215006, P.R. China; 5 Key Laboratory of Thrombosis and Hemostasis of Ministry of Health, The First Affiliated Hospital of Soochow University, Suzhou, Jiangsu 215006, P.R. China; 6 Jiangsu Institute of Clinical Immunology, Suzhou, Jiangsu 215006, P.R. China

**Keywords:** pancreatic cancer, Wnt/β-catenin signal pathway, PP2A, phosphorylation

## Abstract

Cantharidin is an active constituent of mylabris, a traditional Chinese medicine, and presents strong anticancer activity in various cell lines. Cantharidin is a potent and selective inhibitor of serine/threonine protein phosphatase 2A (PP2A). Our previous studies revealed the prospect of application of cantharidin, as well as other PP2A inhibitors, in the treatment of pancreatic cancer. However, the mechanisms involved in the anticancer effect of PP2A inhibitors have not been fully explored. The Wnt/β-catenin pathway is involved in cell migration and proliferation and participates in the progression of pancreatic cancer. If β-catenin is phosphorylated and degraded, the Wnt/β-catenin pathway is blocked. PP2A dephosphorylates β-catenin and keeps the Wnt/β-catenin pathway active. In the present study, we found that PP2A inhibitor treatment induced phosphorylation and degradation of β-catenin. The suppression on the migration and growth of PANC-1 pancreatic cancer cells could be attenuated by pretreatment with FH535, a β-catenin pathway inhibitor. Microarray showed that PP2A inhibitor treatment induced expression changes in 13 of 138 genes downstream of the β-catenin pathway. Real-time PCR further confirmed that FH535 attenuated the expression changes induced by PP2A inhibitors in 6 of these 13 candidate genes. These 6 genes, VEGFB, Dkk3, KRT8, NRP1, Cacnalg and WISP2, have been confirmed to participate in the migration and/or growth regulation in previous studies. Thus, the phosphorylation- and degradation-mediated suppression on β-catenin participates in the cytotoxicity of PP2A inhibitors. Our findings may provide insight into the treatment of pancreatic cancer using a targeting PP2A strategy.

## Introduction

Pancreatic cancer is one of the most fatal solid malignancies, with a 5-year survival rate of only ~5%. There is no method for early detection of the cancer and most patients with localized cancer have no recognizable symptoms. As a result, most patients are not diagnosed until the cancer metastasizes to other organs (1). Less than 20% of patients are eligible for curative resection and, of those, most experience recurrence of the cancer. Thus, an effective treatment and therapy are essential (2).

Mylabris is the dried body of the Chinese blister beetle. The use of mylabris as a traditional Chinese medicine in the treatment of tumor can be traced back to >2,000 years ago and it is still used as a folk medicine today (3). The active constituent of mylabris is cantharidin (3). In our previous studies, we found that cantharidin presented cytotoxicity through NF-κB pathway-mediated apoptosis induction (4), JNK pathway-dependent growth inhibition (3,5) and suppression on migration in breast cancer cells (6). It remains unclear whether cantharidin, as well as other PP2A inhibitors, could suppress the migration of pancreatic cancer cells. Moreover, the mechanism involved in the migration inhibition induced by cantharidin remains unknown.

The conserved Wnt/β-catenin pathway regulates stem cell pluripotency and cell fate decisions during development (7,8). Previous studies presented the participation of Wnt/β-catenin pathway in cell migration and proliferation (9). In the absence of Wnt signal, β-catenin is phosphorylated by glycogen synthase kinase-3β (GSK-3β) and then degraded by the ubiquitin-proteasome system. When Wnt ligands bind to frizzled receptors, GSK-3β activity is inhibited and unphosphorylated β-catenin accumulates in the cytoplasm and translocates into the nucleus, where it acts as a transcription cofactor with T cell factor/lymphoid enhancer factor TCF/LEF (9) and regulates the transcription of a variety of the target genes (7,8). Dysregulation of Wnt/β-catenin signaling and altered transcription of β-catenin/TCF-regulated genes are found in many cancers (10), including pancreatic cancer (11).

Mechanistically, cantharidin has been shown to be a potent and selective inhibitor of serine/threonine protein phosphatase 2A (PP2A) (3). Our previous studies revealed that cantharidin, as well as other PP2A inhibitors, fulfilled their anticancer effect through inhibition of PP2A and subsequent activation of several kinase pathways (3–6). A previous study proved that PP2A dephosphorylates β-catenin. Treatment of colon cancer cells with classical PP2A inhibitor, okadaic acid (OA), increases the phosphorylation of β-catenin (12).

Therefore, in the present study, we investigated whether cantharidin, as well as other PP2A inhibitors, could suppress the migration and growth of pancreatic cancer cells through phosphorylation and degradation of β-catenin-mediated inhibition on the Wnt/β-catenin pathway.

## Materials and methods

### Cells and cultures

The human pancreatic cancer cell line PANC-1 was purchased from the American Type Culture Collection (ATCC; Manassas, VA, USA). Cells were maintained in DMEM medium (Gibco, Grand Island, NY, USA). Medium was supplemented with 10% fetal calf serum (Gibco), 100 U/ml penicillin and 100 mg/ml streptomycin at 37°C in a 5% CO_2_ incubator with humidified atmosphere. Cells were passaged every 2–3 days for exponential growth.

### Reagents

Cantharidin, OA and SP600125 were purchased from Enzo Life Sciences International (Plymouth Meeting, PA, USA). Norcantharidin (NCTD) was purchased from Sigma (St. Louis, MO, USA). FH535 was purchased from Millipore (Billerica, MA, USA).

### Wound healing assay

The cells were seeded in 96-well plates at a density of 1×10^4^ cells/well and grown to confluence. The monolayer culture was then artificially scrape-wounded with a sterile micropipette tip to create a denuded zone (gap) of constant width. Each well was washed with phosphate-buffered saline (PBS) twice to remove the detached cells before treatment. Cells that had migrated to the wounded region were observed using an XDS-1B inverted microscope (MIC Optical & Electrical Instrument, Chongqing, China) and photographed (magnification, ×40). Images were captured at various time points to monitor the wound healing process. The wound areas were measured using ImageJ (NHI, Bethesda, MA, USA).

### MTT assay

Cellular growth was evaluated by the 3-[4,5-dimethylthiazol-2-yl]-2,5-diphenyl tetrazolium bromide (MTT) assay (13). The cells were seeded into 24-well tissue culture plates at 5×10^4^ cells/well. After treatment, MTT (Sigma) was added to each well at a final concentration of 0.5 mg/ml, followed by incubation at 37°C for 4 h. The medium was then removed and 800 μl dimethyl sulfoxide was added to each well. The absorbance of the mixture was measured at 490 nm using a microplate ELISA reader (Bio-Rad Laboratories, Hercules, CA, USA). The relative cell viability was calculated as follows: relative cell viability = (mean experimental absorbance/mean control absorbance) ×100%.

### Western blot analysis

Total protein was extracted using a lysis buffer containing 50 mM Tris-HCl (pH 7.4), 150 mM NaCl, 1% Triton X-100, 0.1% SDS, 1 mM EDTA, supplemented with protease inhibitor cocktail kit (Roche, Indianapolis, IN, USA) and phosphatase inhibitor cocktail kit (Roche). The cytosol and nuclear extracts were prepared by NE-PER nuclear and cytoplasmic extraction reagents (Pierce Biotechnology, Rockford, IL, USA), supplemented with protease inhibitor cocktail kit (Roche). The protein extract was loaded, size-fractionated by SDS-polyacrylamide gel electrophoresis and transferred to PVDF membranes (Bio-Rad Laboratories). After blocking, the membranes were incubated with primary antibodies at 4°C overnight. Rabbit anti-phospho-β-catenin (Thr41/Ser45) antibody was purchased from Santa Cruz Biotechnologies (Santa Cruz, CA, USA). Mouse anti-active-β-catenin (unphosphorylated Ser37/Thr41, clone 8E7) antibody was purchased from Millipore. Mouse anti-β-catenin was purchased from Cell Signaling Technology (Beverly, MA, USA). Rabbit anti-RPL38 and rabbit anti-histone H1 antibodies were purchased from Proteintech Group (Chicago, IL, USA). The protein expression was determined using horseradish peroxidase-conjugated antibodies followed by enhanced chemiluminescence (ECL; Millipore) detection. The intensity of the bands was captured by JS-1035 image analysis scanning system (Peiqing Science & Technology, Shanghai, China). RPL38 and histone H1 were used as internal controls for the total and nuclear extracts, respectively.

### Microarray assay

Sample preparation and processing procedure were performed as described in detail in the Agilent GeneChip Expression Analysis Manual (Santa Clara, CA, USA). Differentially expressed genes were screened using Agilent 44K human whole-genome oligonucleotide microarrays. The selection criterion was defined as a >1.5-fold difference in the level of expression (difference in upregulated expression >1.5-fold and difference in downregulated expression <0.67-fold). Hierarchical clustering of samples was performed by average linkage algorithm using TIGR MultiExperiment Viewer (The Institute for Genomic Research, Rockville, MD, USA).

### Real-time PCR

Total RNA was extracted using TRIzol reagent (Life Technologies) according to the manufacturer’s protocol. After spectrophotometric quantification, 1 μg total RNA was used for reverse transcription in a final volume of 20 μl with AMV reverse transcriptase (Promega) according to the manufacturer’s instructions. Aliquots cDNA of corresponding to equal amounts of RNA were used for the quantification of mRNA by real-time PCR using the LightCycler 96 real-time quantitative PCR detection system (Roche). The reaction system (25 μl) contained the corresponding cDNA, forward and reverse primers and SYBR-Green PCR Master Mix (Roche). All data were analyzed using RPL38 gene expression as an internal standard. The specific primers are presented in Table I.

### Statistical analysis

Each experiment was performed at least in triplicate. Results are expressed as the mean value ± standard deviation (SD). Statistical analysis was performed using an unpaired Student’s t-test. A P-value <0.05 was considered to indicate a statistically significant difference.

## Results

### Cantharidin suppresses cell migration through JNK pathway-independent manner

**Figure 1:**
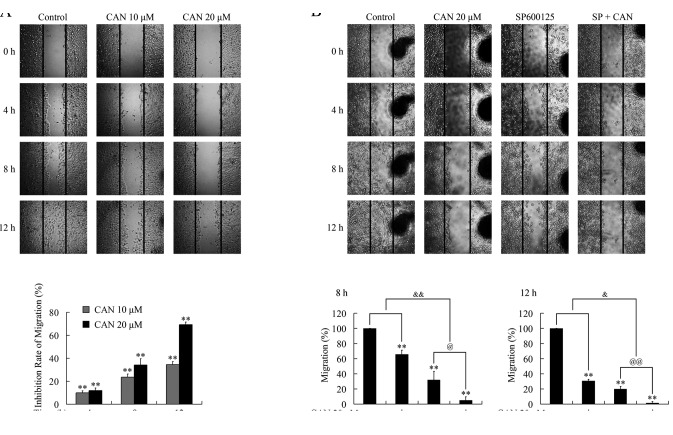
Cantharidin (CAN) suppresses PANC-1 cell migration. (A) CAN suppressed the migration of PANC-1 cells in a time- and dose-dependent manner. ^**^P<0.01, significant differences from the control group. (B) Pretreatment with SP600125 (SP) strengthened the suppression on migration induced by CAN. ^**^P<0.01, significant differences from the respective control groups. ^@@^P<0.01 vs. SP600125 group. ^&&^P<0.01, significant differences between fold inductions.

Previously, we found that cantharidin suppressed the proliferation of pancreatic cancer cells (3,5). However, whether cantharidin affects the migration of pancreatic cancer cells remains unknown. Thus, we investigated whether cantharidin suppresses the migration of pancreatic cancer PANC-1 cells using a wound healing assay. As shown in Fig. 1A, cantharidin suppressed the migration of PANC-1 cells in a time- and dose-dependent manner, suggesting the anticancer effect of cantharidin also involves the suppression on metastasis potential.

In our previous studies, cantharidin-induced activation of JNK suppressed pancreatic cancer cell growth (3,5). As the JNK pathway is also involved in the migration regulation (14), we investigated whether cantharidin suppressed migration of PANC-1 cells through activation of the JNK pathway. Treatment with SP600125, the inhibitor of JNK, time-dependently suppressed migration of PANC-1 cells, suggesting the basal activity of the JNK pathway promoted migration of pancreatic cancer cells. Notably, pretreatment with SP600125 did not attenuate the cantharidin-induced suppression on migration, but even strengthened the cantharidin-induced suppression of cell migration (Fig. 1B), suggesting the activation of JNK impaired the migration suppression of cantharidin.

### PP2A inhibitors suppress the β-catenin pathway through phosphorylation-mediated degradation of β-catenin

**Figure 2:**
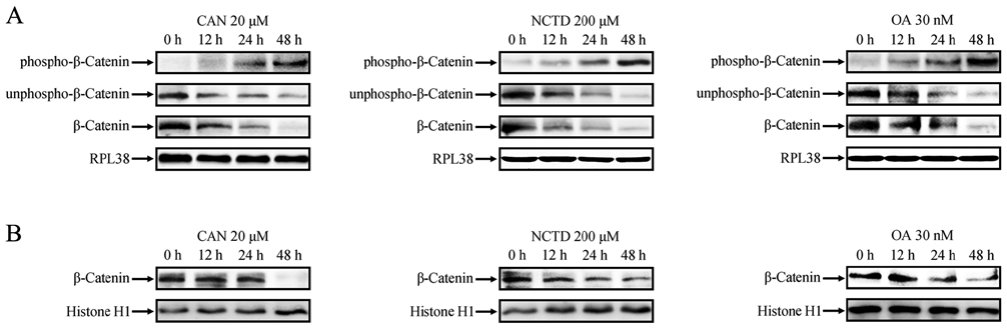
Treatment of PP2A inhibitors suppresses β-catenin pathway. (A) Treatment with PP2A inhibitors, CAN, NCTD and OA, increased the phosphorylation level, decreased the nonphosphorylated form (active form) of β-catenin and reduced total protein level of β-catenin. (B) Treatment with PP2A inhibitors decreased nuclear distribution of β-catenin. PP2A, protein phosphatase 2A; CAN, cantharidin; NCTD, norcantharidin; OA, okadaic acid.

Treatment with PP2A inhibitors, cantharidin, NCTD and OA, increased the phosphorylation level and decreased the nonphosphorylated form (active form) of β-catenin, accompanied by the reduction of total protein level of β-catenin (Fig. 2A). Blotting of nuclear protein proved the decreased nuclear distribution of β-catenin after treatment with PP2A inhibitors (Fig. 2B). These data suggested that PP2A inhibitors induced phosphorylation and degradation of β-catenin, which further resulted in the decreased nuclear translocation.

### PP2A inhibitors suppress migration and growth of pancreatic cancer cells through suppression on β-catenin pathway

**Figure 3:**
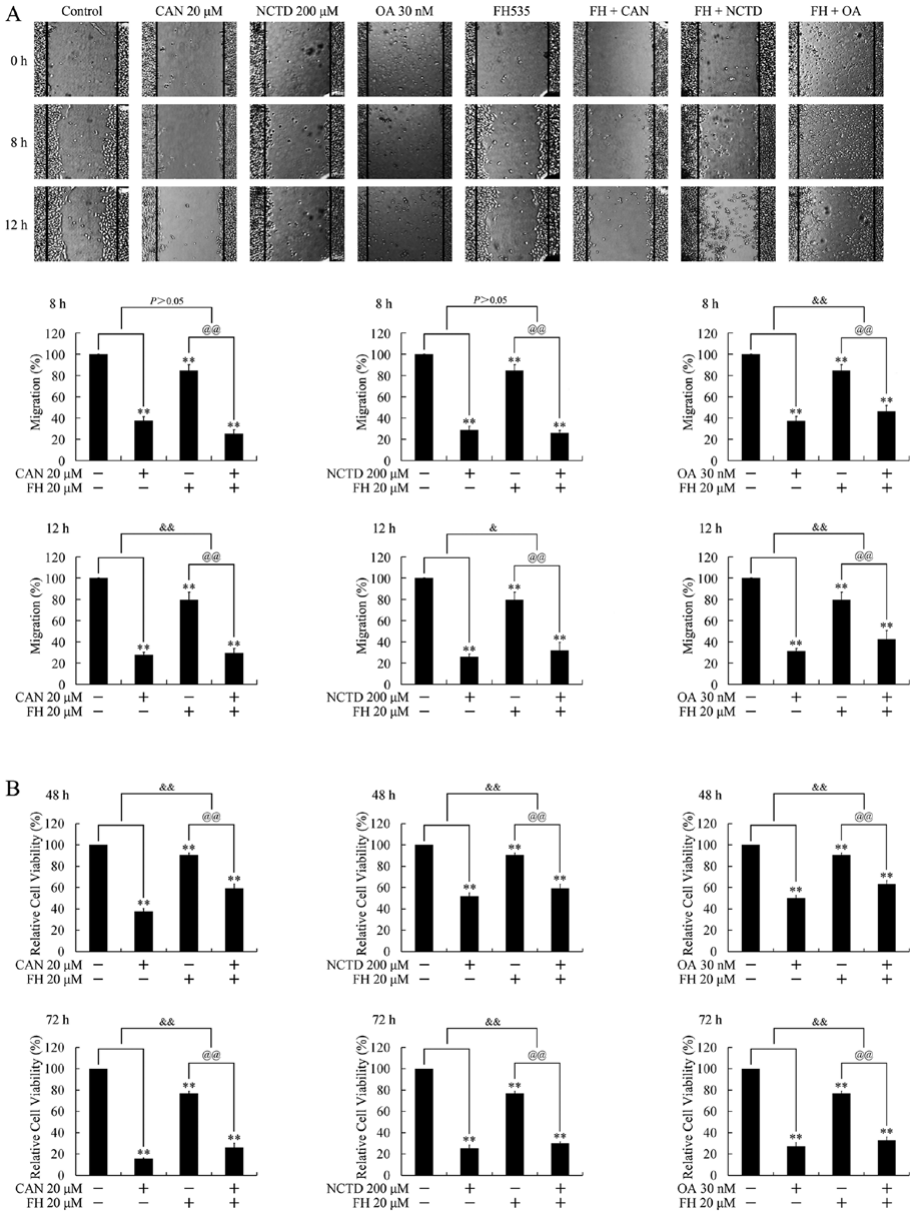
Pretreatment of PANC-1 cells with β-catenin pathway inhibitor attenuates the migration and growth inhibition induced by PP2A inhibitors. (A) Treatment with FH535 (FH), CAN, NTCD or OA suppressed the migration of PANC-1 cells. Pretreatment with FH535 attenuated the migration inhibition induced by CAN, NTCD or OA. (B) Treatment with FH535, CAN, NTCD or OA suppressed the growth of PANC-1 cells. Pretreatment with FH535 attenuated the growth inhibition induced by CAN, NTCD or OA. ^**^P<0.01, significant differences from the respective control groups. ^@@^P<0.01 vs. FH535 group. ^&&^P<0.01, significant differences between fold inductions. CAN, cantharidin; NCTD, norcantharidin; OA, okadaic acid.

To investigate whether the suppression on the β-catenin pathway is involved in the anticancer effect of PP2A inhibitors, we used FH535, a classic Wnt/β-catenin inhibitor (15), to verify whether blocking Wnt/β-catenin could attenuate the migration and growth suppression caused by PP2A inhibitors. Treatment with FH535 suppressed the migration and growth of pancreatic cancer cells, suggesting β-catenin may participate in the progression of pancreatic cancer (Fig. 3A and B).

Cantharidin, NCTD and OA suppressed the migration and growth of pancreatic cancer cells, respectively. When cells were pretreated with FH535, the anti-migration and anti-growth effects of PP2A inhibitors were partially attenuated, suggesting that the anticancer effects of PP2A inhibitors were partially β-catenin pathway-dependent.

### PP2A inhibitors induce expression changes of genes at the downstream of the β-catenin pathway

**Figure 4:**
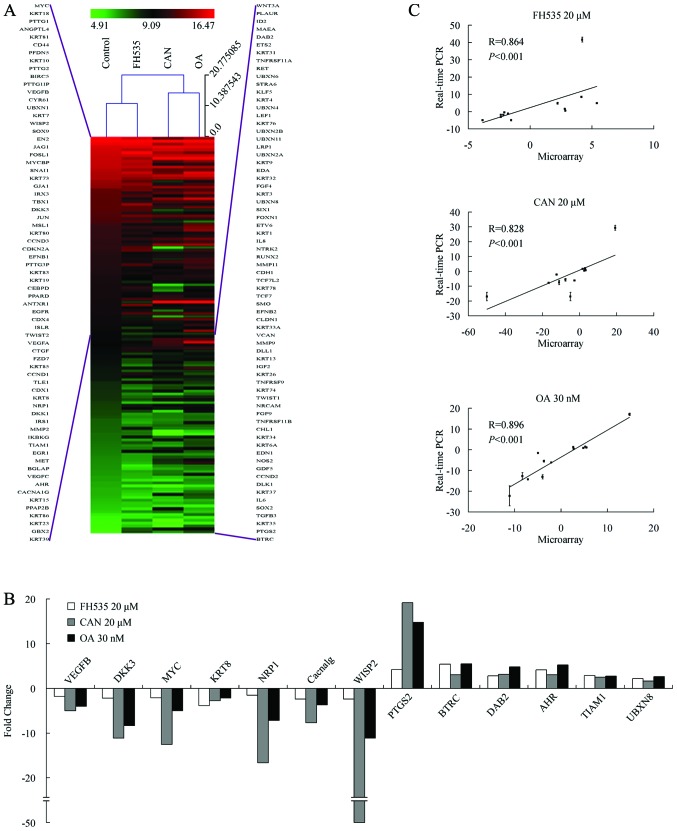
Regulation of expression of genes downstream of the Wnt/β-catenin pathway upon treatment with PP2A inhibitors. (A) Illustration of results of the microarrays. (B) Expression change folds of genes shared the same tendency when treated with FH535 or PP2A inhibitors. (C) Validation of the microarray results on 13 genes by real-time PCR using Spearman’s rank correlation analysis. PP2A, protein phosphatase 2A.

We performed microarray analyses to determine the mRNA expression changes of 138 genes downstream of β-catenin pathway (http://www.stanford.edu/group/nusselab/cgi-bin/wnt/target_genes) (Fig. 4A). Only those genes that significantly altered expression levels (by 1.5-fold) in all FH535, cantharidin and OA groups were chosen for further analysis. Of the 138 genes analyzed, 13 fulfilled this criterion (Fig. 4B).

We used real-time PCR to confirm the changes of these genes in the microarray analysis. Spearman’s rank correlation analysis revealed that the mRNA expression values obtained by real-time PCR correlated significantly with those obtained by microarray analysis for these 13 genes (P<0.01). The correlation coefficient R ranged between 0.828 and 0.896 (Fig. 4C).

### PP2A inhibitors induce expression level changes of candidate genes through β-catenin pathway-dependent manner

**Figure 5:**
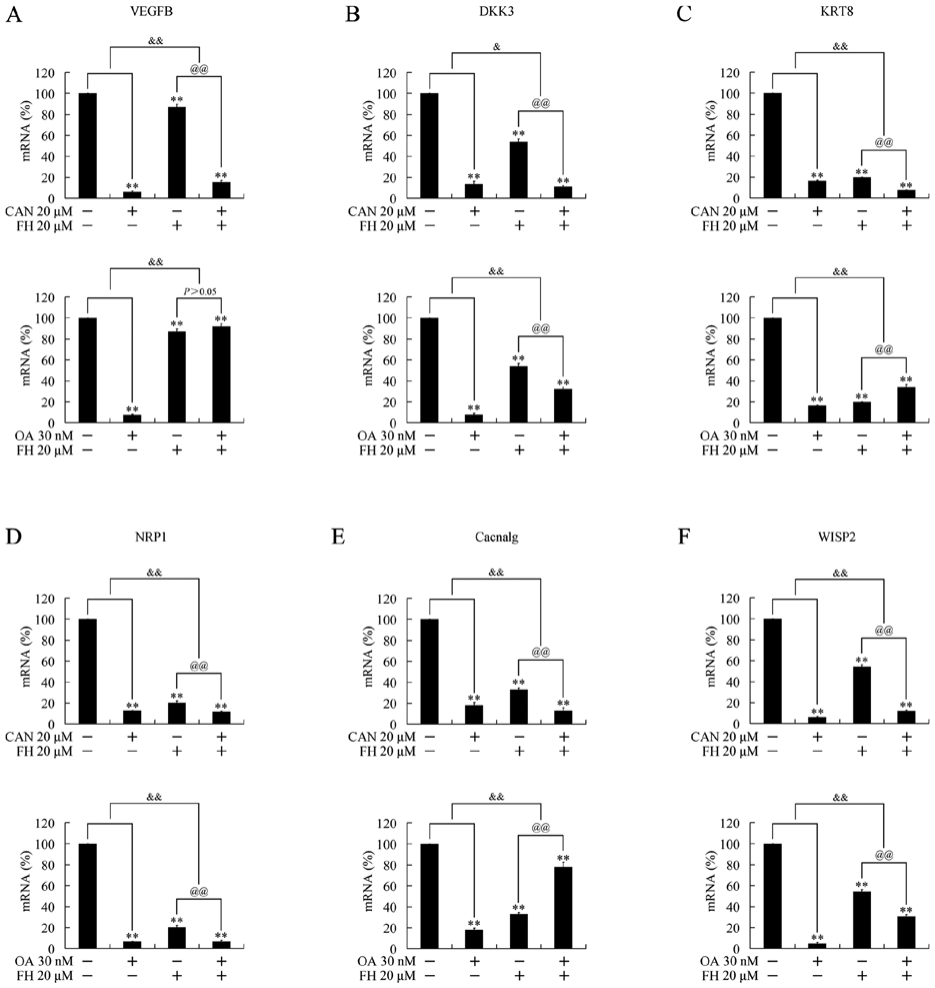
PP2A inhibitors induce expression changes of VEGFB, Dkk3, KRT8, NRP1, Cacnalg and WISP2 through β-catenin pathway-dependent manner in PANC-1 cells. Pretreatment of PANC-1 cells with FH535 attenuated or reversed the downregulation of VEGFB (A), Dkk3 (B), KRT8 (C), NRP1 (D), Cacnalg (E) and WISP2 (F) triggered by PP2A inhibitors. ^**^P<0.01, significant differences from the respective control groups. ^@@^P<0.01 vs. FH535 group. ^&^P<0.05 and ^&&^P<0.01, significant differences between fold inductions. PP2A, protein phosphatase 2A.

To verify whether the expression changes of these 13 genes induced by PP2A inhibitors were executed through β-catenin pathway-dependent manner, the cells were pretreated with FH535, followed by treatment with PP2A inhibitors. As shown in Fig. 5, expression changes of 6 of these 13 genes, VEGFB, Dkk3, KRT8, NRP1, Cacnalg and WISP2, were attenuated by FH535 pretreatment, suggesting PP2A inhibitors downregulated these 6 genes in a β-catenin pathway-dependent manner.

## Discussion

The Wnt/β-catenin pathway has been positively confirmed to regulate cell proliferation, migration, apoptosis, differentiation and stem cell self-renewal (16,17). As the indispensable mediator of classical Wnt signaling pathway, β-catenin participates in two distinct functions in the cell, depending on its localization. Membrane-localized β-catenin is isolated by the epithelial cell-cell adhesion protein E-cadherin to chronically maintain cell-cell adhesion (9). On the other hand, classical Wnt signaling pathway causes accumulation of β-catenin in cytoplasm in complex with the transcription factor TCF/LEF that regulates target gene expression (18,19). In the absence of Wnt signaling, the level of β-catenin is kept low through degradation of cytoplasmic β-catenin, which is targeted for phosphorylation and ubiquitination at Ser33/Ser37/Thr41 by GSK3β bound to a scaffolding complex of axin and adenomatous polyposis coli (APC) protein (20,21). Thus, the key factors in β-catenin signaling are its stabilization and accumulation in the cytoplasm.

This is a reflection of an evolving literature showing Wnt/β-catenin signaling has variable and sometimes paradoxical effects in the pancreas dictated by its timing, location, strength and mechanism of activation. Several investigators have demonstrated that β-catenin was essential for normal pancreatic development through the canonical Wnt signaling pathway, but this pathway is downregulated in adult pancreas (22). Although canonical activating mutations are uncommon, Wnt/β-catenin signaling can be dysregulated in pancreatic cancer through a variety of mechanisms that modulate existing levels of autocrine or paracrine Wnt activation. There is ample *in vitro* and *in vivo* evidence that Wnt/β-catenin signaling is involved in pancreatic cancer tumorigenesis. Aberration in canonical Wnt/β-catenin signaling activity has been documented in pancreatic cancer (23). Positive expression of nuclear and/or cytoplasmic β-catenin is reported in anywhere from 4 to 65% of human pancreatic ductal adenocarcinoma tumors (11) and up to 40% of pancreatic intraductal papillary mucinous neoplasms (24). Positive nuclear β-catenin distribution is also reported in advanced pancreatic intraepithelial neoplasia in human and mouse model (25,26). Retrospective studies reported alterations in β-catenin that correlate with tumor differentiation (27,28) metastasis (29,30) or patient survival (30,31). Heiser *et al* (32) demonstrated that enhanced Wnt/β-catenin signaling in itself could induce pancreatic tumorigenesis and activation of other oncogenes in the presence of enhanced Wnt/β-catenin signaling induced distinct pancreatic tumor formation. This dysregulation makes it evident that these changes have meaningful phenotypic effects on pancreatic cancer tumorigenesis. The direct inhibition of Wnt/β-catenin signaling by knockdown of β-catenin suppresses human pancreatic cancer cell growth and survival *in vitro* (26). Contrary to colon cancer, in which the genetic mutations are common, the manner in which Wnt/β-catenin signaling is activated and readily modulated in pancreatic cancer may also indicate that pancreatic cancer may be more amenable to genetic or pharmacological targeting of Wnt/β-catenin as clinical therapy (11).

β-catenin is the first identified target of PP2A-B56α. Overexpression of B56α decreased β-catenin expression in mammalian cells and Xenopus embryo explants (33). PP2A-B56α is thought to have targets within Axin1-mediated degradation complex for β-catenin and it has been found to be able to inhibit the Wnt signaling pathway (34). A previous study proved that the effect of aspirin on the Wnt/β-catenin pathway is mediated via PP2A (12). Aspirin treatment caused increased phosphorylation of Tyr307 of PP2A, an event associated with inhibition of PP2A enzymatic activity. Inhibition of PP2A resulted in phosphorylation of β-catenin and inhibition of β-catenin/TCF transcriptional activity. Although the phosphorylation-mediated degradation of β-catenin was not observed in this literature, these findings provided a molecular explanation for the efficacy of aspirin in chemoprevention of colorectal cancer and shows biochemical evidence that PP2A is an important regulator of Wnt/β-catenin pathway activity in colorectal cells. In our present study, PP2A inhibitors triggered phosphorylation and degradation of β-catenin in pancreatic cancer cells, suggesting inhibition of β-catenin pathway induced by inhibition of PP2A could be a promising way in cancer treatment.

To demonstrate whether cantharidin and other PP2A inhibitors suppress pancreatic cell migration by phosphorylation/degradation of β-catenin and alter expressions of genes downstream of the Wnt/β-catenin pathway, we firstly treated the pancreatic cancer cells with cantharidin and other PP2A inhibitors, and evaluated the migration and growth of the cells. Then, we determined the phosphorylation and protein levels of β-catenin and expression level changes of genes downstream of the Wnt/β-catenin pathway. We found that cantharidin and other PP2A inhibitors suppressed the migration of pancreatic cells through the Wnt/β-catenin pathway by phosphorylation/degradation of β-catenin. By using genome microarray technology and RT-PCR, we identified 6 candidate genes, VEGFB, NRP1, Dkk3, KRT8, Cacnalg and WISP2, at the downstream of PP2A/β-catenin pathway.

VEGFB is a prototype member of vascular endothelial growth factor (VEGF) family, which participates in both physiologic and pathologic angiogenesis (35). VEGFB has been found to be able to promote migration and invasion, but not proliferation or survival in pancreatic cancer cells (36). NRP1 has a protumorigenic role and direct contribution to tumor progression in some studies where NRP1 is predominantly expressed in epithelial cancer cells, including carcinomas of lung, breast, prostate, pancreas and colon and is implicated in the survival, migration and invasion of tumor cells (37–40). NRPl overexpression is positively associated with metastatic potential, advanced stage, shorter 5-year survival rate and/or clinical grade in prostate (41), gastrointestinal (42) and colorectal carcinoma (43), suggesting a protumorigenic role of NRP1 and direct contribution of NRP1 to tumor progression. Dkk3 is involved in embryonic development through its interactions with the Wnt signaling pathway. Dkk3 maintains the pancreatic cancer cells in a dedifferentiated state. Knockdown of Dkk3 resulted in significant reduction of cellular proliferation and concomitant induction of cell cycle inhibitors, as well as pancreatic epithelial cell differentiation markers (44). KRT8 (Keratin 8) is a member of the type II keratin family and knockdown of KRT8 increased migration and invasiveness, but increased apoptosis in epithelial cancer cells through upregulation of Fas receptor (45). Cacnalg (Ca^2+^ channel, voltage-dependent, T-type, α 1G subunit) is expressed in various human tumors, including colon and pancreatic cancer and glioblastoma, as well as in acute myelogenous leukemia (46,47). Knockdown of Cacnalg decreased proliferation of astrocytoma, neuroblastoma, renal tumor (48), breast cancer (49) and glioblastoma (50) cells. WISP2 (Wnt-induced secreted proteins 2), one of the three Wnt inducible proteins that belongs to the CCN family, stimulates mitosis, adhesion, apoptosis, extracellular matrix production, growth arrest and migration of multiple cell types (51). Previous findings suggest that WISP2 is relevant to tumorigenesis and malignant transformation. Its roles in cancer appear to vary depending on cell type and the microenvironment (52) and promote invasiveness and growth in the circumstance of pancreatic cancer (53). As VEGFB, NRP1, Dkk3, KRT8, Cacnalg and WISP2 all participate in the migration and/or growth regulation, the PP2A/β-catenin pathway-mediated migration and growth inhibition induced by PP2A inhibitors could be executed through these 6 genes.

PP2A is generally considered to be a cancer suppressor. Inhibition of PP2A has been thought to be cancer promoting by induction of phosphorylation and activation of several substrate kinases, including c-Jun N-terminal kinase (JNK), extracellular signal-related kinase (ERK), p38, Akt and protein kinase C (PKC) amongst others, most of which can accelerate growth (54–55). However, we previously reported some kinase-dependent anticancer pathways that are induced by treatment with PP2A inhibitors, which suggested that the activation of kinase pathways may not always be cancer promoting. These kinase-dependent anticancer mechanisms induced by PP2A inhibitors include JNK-dependent growth inhibition (5), NF-κB pathway-dependent apoptosis induction (4) and PKC-dependent downregulation of α2 integrin (6). In the present study, we confirmed that the phosphorylation-mediated inhibition of the β-catenin pathway participated in the migration and growth inhibition effect of PP2A inhibitors.

The JNK pathway, over-activation of which suppresses the growth of pancreatic cancer cells (5), fulfilled its positive effect on migration upon treatment with PP2A inhibitors. A similar phenomenon was also found in our previous studies. The activation of PKC decreased the expression of intergrin and suppressed the adhesion to platelet, which suppress the metastasis potential. However, the activation of PKC impaired the growth inhibition effect of PP2A inhibitors. These may be ascribed to the multiple-targeting effects of PP2A, i.e., an inhibition of PP2a may cause dysfunction of abundant pathways of PP2A and the anticancer effect of PP2A inhibitors is a joint function of several pathways. These also suggest that a signaling pathway may present opposite effects in different cellular processes, which may also be affected by several signal pathways.

In conclusion, we demonstrated that PP2A inhibitors suppressed pancreatic cancer cell migration and growth through the Wnt/β-catenin pathway by phosphorylation and further degradation of β-catenin. This may be attributed to the selective downregulation of genes downstream of the Wnt/β-catenin pathway. Our findings provide a promising strategy for treatment of pancreatic cancer by targeting PP2A using PP2A inhibitors.

## Figures and Tables

**Table I:** Primers.

Genes	Sense (5′-3′)	Antisense (5′-3′)	Product size (bp)
VEGFB	GGACAGAGTTGGAAGAGGAGAC	GGGAGGAAGAGCCAGTTGTA	130
Dkk3	GGGAGGAGATGGAAACAATG	ATGGAAAGAACTGCGTGGAA	131
MYC	CCTCCACTCGGAAGGACTATC	TTGTGTGTTCGCCTCTTGAC	142
KRT8	CAGGAGCTGATGAACGTCAA	TCATGTTCTGCATCCCAGAC	106
NRP1	GACAGAAACTGGATGCCTGAA	CTTCCCACCCTGAATGATGA	153
Cacnalg	CACTCTCTGCCCAATGACAG	ACAAGACGGAGCCTGACTGA	109
WISP2	ACTCCCTGCCTACACACACAG	TGCCTTCTCTTCATCCTACCC	165
PTGS2	TAGGTGCATTGGAATCAAGC	GGAGAAACGAAGTGATGAGAAGA	105
BTRC	GCAGTCCAACCCAGATTAGTG	AAATGGCTCTCTTTCCGATACT	135
DAB2	TGTGGCTTCTTCTCAACCTG	TTATTCCTCTGGATGGTCTGC	104
AHR	ACGAGGTCAAGAGATGGAGAC	TTCCCAGGTTCAGGCTATTC	148
TIAM1	CTTCCCTCATCCCAGCAATA	CCTCCTCCTCCCAAGAGACT	120
UBXN8	GCAGCAAAGAGCCAGAACTT	GGACATCGGAGAGCAACAGT	143
RPL38	GCTGCTTGCTGTGAGTGTCT	AGATTTGGCATCCTTTCGTC	149
